# Cardiovascular Disease among Breast Cancer Survivors

**DOI:** 10.31487/j.cdm.2020.01.01

**Published:** 2020-03-06

**Authors:** Steven S. Coughlin, Deepak Ayyala, Ban Majeed, Letisia Cortes, Gaston Kapuku

**Affiliations:** 1 Department of Population Health Sciences, Medical College of Georgia, Augusta University, Augusta, GA, USA; 2 Institute of Public and Preventive Health, Augusta University, Augusta, GA, USA; 3 Medical College of Georgia, Augusta University, Augusta, GA, USA; 4 Department of Medicine, Medical College of Georgia, Augusta University, Augusta, GA, USA

**Keywords:** Breast cancer survivors, cardiomyopathy, cardiovascular disease, congestive heart failure, hypertension, stroke, venous thrombosis

## Abstract

**Background:**

Among breast cancer survivors age > 50 years, deaths due to cardiovascular disease account for 35% of non-cancer related deaths. The increases in cardiovascular disease among breast cancer survivors is due to the cardiotoxic effects of breast cancer treatment and to overlapping risk factors for breast cancer and cardiovascular disease.

**Methods:**

We conducted a study of a sample of 164 breast cancer patients in order to examine the frequency of cardiovascular disease. The overall objective was to examine the frequency of high blood pressure, myocardial infarction, cardiomyopathy, congestive heart failure, stroke, and venous thrombosis/thromboembolism among women who have been diagnosed with stage I-IV breast cancer and who had completed primary therapy for the disease. Data were collected by postal survey and abstraction of electronic medical records.

**Results:**

A high percentage of the women (62.8%) had a reported history of high blood pressure. Fifty percent of the women had a reported history of high cholesterol. About 8.3% of the women were current smokers and 36.0% were former smokers. About 23.8% of the women had a reported history of diabetes. About 4.9% of the women had a reported history of congestive heart failure and 6.1% had a history of stroke.

**Discussion:**

Additional studies are needed of cardiovascular risk factors and adverse cardiovascular events among breast cancer survivors. Of particular concern is whether patients with hypertension, hypercholesterolemia, and diabetes are receiving appropriate therapy to reduce their cardiovascular risk and prevent morbidity and mortality from adverse cardiovascular events.

## Introduction

Cardiovascular disease is the leading cause of death in women in general and among breast cancer survivors in particular [1, 2]. Among breast cancer survivors aged fifty and more, deaths due to cardiovascular disease account for 35% of non-cancer related deaths [1]. Cardiovascular disease is the leading cause of death in patients with breast cancer over fifty years of age [3]. The increases in cardiovascular disease among breast cancer survivors is due to the cardiotoxic effects of breast cancer treatment (e.g. anthracycline chemotherapy and biologic therapy) and to overlapping risk factors for breast cancer and cardiovascular disease (e.g. age, physical inactivity, obesity, insulin resistance, alcohol consumption, hormone replacement therapy) [4]. There is a well-established link between anthracycline chemotherapy and cardiomyopathy and congestive heart failure [1, 2].

As a result, doses of anthracycline such as doxorubicin have decreased. Trastuzumab, a biological therapy that targets the HER-2 receptor can lead to reversible congestive heart failure [5-7]. Tamoxifen used as an adjuvant therapy for breast cancer increases risk of venous thrombosis [2]. We conducted a study of a sample of breast cancer patients in order to examine the frequency of cardiovascular disease. The overall objective was to examine the frequency of congestive heart failure, high blood pressure, myocardial infarction, cardiomyopathy, stroke, and venous thrombosis/thromboembolism among women who have been diagnosed with stage I-IV breast cancer and had completed primary therapy for the disease. The specific aim was to examine the relationship between congestive heart failure and cancer treatment with chemotherapy.

## Methods

Data are from the Cardiovascular Disease outcomes among Breast Cancer Survivors study (CVDBCS), a cross-sectional study among breast cancer survivors. Non-institutionalized women were eligible to take part in the study if they had been diagnosed with breast cancer and completed primary therapy at Augusta University Health or the Georgia Cancer Center; and resided in Augusta-Richmond County or Columbia County, GA, or in Aiken County, SC. Data were collected using postal survey questionnaires and abstraction of electronic medical records.

The mailings were sent to 1,000 potential research participants. A sequential mailing protocol was followed using a modified Dillman method. An advance letter was mailed to the women by the study principal investigator (SSC). The letter provided information about the study (purpose, potential benefits, and risks). Three weeks later, a survey consent letter was mailed to those who had not opted out along with a copy of the survey questionnaire and a pre-addressed, stamped return envelope. Women who had not opted out or returned a completed questionnaire were sent a reminder postcard four weeks later.

## Outcome Measures

Information about cardiovascular risk factors and diseases (congestive heart failure, high blood pressure, diabetes, myocardial infarction, cardiomyopathy, stroke, venous thrombosis or thromboembolism), breast cancer (stage-at-diagnosis, date of diagnosis), and breast cancer treatment) was collected via postal survey and abstracted from electronic medical records. Survey responses were checked for completeness and then coded and entered into an electronic database.

Descriptive analyses and logistic regression methods were used to examine predictors of congestive heart failure, using a forward-selection model building strategy. We present adjusted odds ratios (OR) and 95% confidence Intervals (95% CI). Significance level was set at α = 0.05. Levels of statistical significance were determined using Wald chi-square tests and Log-likelihood ratio tests. The goodness-of-fit of the model model was examined using the Log-likelihood ratio test.

## Result

A total of 164 women completed the study questions (response rate 16.4%). The mean age of the women was 67 years (Table 1). Among all participants, 66.7% were white, 29.5% were African-American, and the remainder were of other races. More than half (58.4%) of the women were insured through Medicare and 29.2% held private insurance. With respect to breast cancer stage at diagnosis, 19.8% of the women had ductal carcinoma in situ, 26.8% had stage I disease, 21.0% had stage II disease, 8.9% had stage II disease, and 5.1% had stage IV disease. The mean number of years since diagnosis was 9.4 years. About 54.9% of the women reported receiving chemotherapy and only 4.9% reported biologic/targeted therapy.

A high percentage of the study participants (62.8%) reported a history of high blood pressure; 50% had high cholesterol; and 8.3% were current cigarette smokers (Table 2). About 23.8% of the women had a reported history of diabetes. About 4.9% of the women had a reported history of congestive heart failure and 6.1% had a history of stroke. Similar results were obtained based upon information recorded in the medical records (Table 3). In a logistic regression analysis of predictors of congestive heart failure, time since diagnosis (years) was a significant predictor of congestive heart failure (odds ratio [OR] = 1.14, 95% confidence interval [CI] 1.03-1.23). Women who had received chemotherapy were more likely to have a history of congestive heart failure (OR = 1.43, 95% CI 0.57-361.67); the association was of borderline significance (p=.1059).

## Discussion

In this study of breast cancer survivors treated at a large academic medical center in the southern United States, a high percentage of patients had one or more cardiovascular risk factors. Almost two-thirds of the women had a history of high blood pressure and half had a history of high cholesterol. About one in five had a history of diabetes. Forty-four percent were current or former cigarette smokers. The prevalence of cardiovascular risk factors in this population is of concern both because it may increase risk of adverse cardiovascular events such as congestive heart failure and stroke, and because breast cancer survivors have an increased risk of adverse cardiovascular events due to primary treatment for the disease. Aside from hypertension, the most common adverse cardiovascular disease outcomes were stroke (5.1%), venous thrombosis (5.1%), congestive heart failure (4.4%), and cardiomyopathy (4.4%). Only 1.3% of the women had a recorded history of heart attack. Breast cancer survivors who receive tamoxifen therapy have an increased risk of deep vein thrombosis and pulmonary embolism [8].

Women who had received chemotherapy were more likely to have a history of congestive heart failure. This is consistent with the well-established link between anthracycline chemotherapy and congestive heart failure [1, 2]. However, we lacked information about the specific chemotherapy regimens received by the patients. Cardiovascular disease is the leading cause of death for women with ductal carcinoma in situ or stage I disease, and for women aged > 80 years with stage II disease [9]. A recent systematic review found that women with a history of breast cancer have a higher risk of breast cancer mortality than their cancer-free counterparts [10]. However, in the National Institute of Environmental Health Sciences Sister Study Cohort, changes over time in cardiovascular disease risk, adiposity measures, and blood pressure were similar between women who developed an incident breast cancer and those who did not [9].

Over half of the patients in the current study were over 60 years of age. Older breast cancer survivors cope with health issues related to cancer treatment and the aging process, including comorbidities, symptoms, physical functioning, nutrition, and physical activity [11]. Additional research is needed to examine therapeutic interventions to address the health conditions older breast cancer survivors are coping with [11]. Cardiotoxicity is a major concern during breast cancer therapy and may impede the normal chemotherapy regimen [1]. Cardiovascular side effects of chemotherapy used in cancer therapy is increasingly becoming a medical problem of paramount importance since the number of cancer survivors is increasing. Common chemotherapy agents such as adriamycin and trastuzumab may induce cardiac malfunction that is reversible after stopping the treatment and/ or using ACE inhibitors and/or beta blockers [1, 2]. Cardiac malfunction is highly prevalent in young breast cancer survivors treated with anthracycline [3].

It is important to develop early markers of cardiotoxicity to prevent irreversible damage. This can be accomplished by detecting early heart function derangement than relying on ejection fraction which is less sensitive to capture subtle heart malfunction. Monitoring diastolic function has been proved to be more sensitive to effect of these medications in the increasing numbers cancer survivors. Such effort will encompass using Doppler and strain rate imaging, cardiac MRI, and biomarkers (e.g. troponin, BNP) in a large population to identify precursors of cardiac malfunction in breast cancer treated patients and determine who should be treated at an early stage.

With respect to limitations, misclassification bias is a possibility due to the use of self-reported information. However, recorded information about cardiovascular risk factors and adverse cardiovascular outcomes was also obtained from electronic medical records. The results of this study may not be generalizable to other populations of breast cancer survivors. However, the sample was diverse by race, socioeconomic factors, and history of breast cancer diagnosis and treatment. Additional studies are needed of cardiovascular risk factors and adverse cardiovascular events among breast cancer survivors. Of particular concern is whether patients with hypertension, hypercholesterolemia, and diabetes are receiving appropriate therapy to reduce their cardiovascular risk and prevent morbidity and mortality from adverse cardiovascular events.

## Figures and Tables

**Table 1:** Characteristics of study participants, Cardiovascular Disease Outcomes among Breast Cancer Survivors Study (n=164).

Characteristic	Frequency (%)
**Age** (years) mean (SD) (N=163)	67 (41.1)
**Race** (N=156)	
White, Non-Hispanic	104 (66.7)
African American, Non-Hispanic	46 (29.5)
Other^1^	6 (3.9)
**Annual Income** (N=46)	
<$20,000	17 (10.4)
$20,000-$34,999	17 (10.4)
$35,000-$49,999	17 (10.4)
$50,000-$64,999	14 (8.5)
$65,000-$79,999	8 (4.9)
$80,000 +	38 (23.2)
Missing^2^	53 (32.3)
**Number of people in household** (N=160)	
1	48 (30.0)
2	83 (51.9)
3 +	29 (18.1)
**Employment status** (N=163)	
Retired	99 (60.7)
Employed	34 (20.9)
On disability	16 (9.8)
Homemaker	9 (5.5)
Temporarily unemployed	4 (2.5)
**Marital status** (N=163)	
Married/Partner	84 (51.5)
Single	24 (14.7)
Widowed	32 (19.6)
Separated/Divorced	23 (14.1)
**Education** (N=157)	
Less than HS	5 (3.2)
HS or equivalent	42 (26.8)
Some college	27 (17.2)
Associate degree	22 (14.0)
Bachelor degree	27 (17.2)
Graduate degree	34 (21.7)
**Health Insurance** (N=161)	
Medicare	94 (58.4)
Private insurance	47 (29.2)
Other^3^	20 (12.4)
**Perceived general health** (N=162)	
Excellent	16 (9.9)
Very good	58 (35.8)
Good	59 (36.4)
Fair	24 (14.8)
Poor	5 (3.1)
**Breast cancer stage at diagnosis** (N=157)	
Ductal carcinoma in situ	31 (19.8)
Stage I	42 (26.8)
Stage II	33 (21.0)
Stage III	14 (8.9)
Stage IV	8 (5.1)
Don’t know	29 (18.5)
**Time since diagnosis** (in years) mean (SD) (N=155)	9.4 (8.8)
**Type of treatment received**^4^ (N=164)	
None	2 (1.2)
Surgery	161 (98.2)
Radiation	111 (67.7)
Chemotherapy	90 (54.9)
Hormone therapy	74 (45.1)
Biologic/Targeted therapy	8 (4.9)

**Table 2:** Self-reported History of Cardiovascular Disease/Risk Factors and among Breast Cancer Survivors (n=164).

Cardiovascular disease/risk factor	Frequency (%)
Heart attack	3 (1.8)
Congestive heart failure	8 (4.9)
Stroke	10 (6.1)
Diabetes	39 (23.8)
High blood pressure	103 (62.8)
High cholesterol	82 (50.0)
Cigarette smoking status	13 (8.3)
Current smoker	
Former smoker	56 (36.0)
Never smoker	87 (55.7)

**Table 3:** Recorded History of Cardiovascular Risk Factors and Cardiometabolic Disease among Breast Cancer Survivors, from Abstraction of Electronic Medical Records (n=164).

Factor	Frequency (%)
High Blood Pressure	102 (64.6)
High Cholesterol	75 (47.5)
Congestive Heart Failure	7 (4.4)
Heart Attack	2 (1.3)
Stroke	8 (5.1)
Cardiomyopathy	7 (4.4)
Venous Thrombosis	8 (5.1)
